# Soybean Meal-Dependent Acute Intestinal Inflammation Delays Osteogenesis in Zebrafish Larvae

**DOI:** 10.3390/ijms23137480

**Published:** 2022-07-05

**Authors:** Marta Carnovali, Giuseppe Banfi, Giovanni Porta, Massimo Mariotti

**Affiliations:** 1 IRCCS Istituto Ortopedico Galeazzi, 20161 Milan, Italy; marta.carnovali@grupposandonato.it (M.C.); giuseppe.banfi@hsr.it (G.B.); 2 School of Medicine, Vita-Salute San Raffaele University, 20132 Milan, Italy; 3 Centro di Medicina Genomica, Department of Medicine and Surgery, University of Insubria, 21100 Varese, Italy; giovanni.porta@uninsubria.it; 4 Department of Biomedical, Surgical and Dental Sciences, University of Milan, 20122 Milan, Italy

**Keywords:** zebrafish, soy, osteogenesis, chondrogenesis, intestinal inflammation

## Abstract

Foods are known to be modulators of inflammation and skeletal development. The intestine plays an essential role in the regulation of bone health mainly through the regulation of the absorption of vitamin D and calcium; in fact, inflammatory bowel diseases are often related to bone health issues such as low bone mineral density, high fracture risk, osteoporosis and osteopenia. Considering the complexity of the pathways involved, the use of a simple animal model can be highly useful to better elucidate the pathogenic mechanisms. Soybean flour with a high saponin content has been used in many studies to induce intestinal inflammation in zebrafish larvae. Using a 50% soybean meal (SBM), we analyzed the effects of this soy-induced inflammatory bowel disease on zebrafish larval osteogenesis. Soybean meal induces intestinal functional alterations and an inflammatory state, highlighted by neutral red staining, without altering the general development of the larvae. Our data show that the chondrogenesis as well as endochondral ossification of the head of zebrafish larvae are not affected by an SBM-diet, whereas intramembranous ossification was delayed both in the head, where the length of the ethmoid plate reduced by 17%, and in the trunk with a delayed vertebral mineralization of 47% of SBM larvae. These data highlight that diet-dependent bowel inflammation can differently modulate the different mechanisms of bone development in different zones of the skeleton of zebrafish larvae.

## 1. Introduction

Foods have been known to modulate human physiology and, consequently, body structures. The skeletal system, for example, is known to be strongly influenced by dietary habits [1]. Foods have also been correlated with inflammatory bowel diseases which, in turn, can generate multiple complications [2,3] including bone issues such as a high risk of low bone mineral density and increased incidence of fractures [4].

The study of the impact of natural products and foods on human health can be conducted *in vivo* with simple animal models which mimic the human physiology, such as zebrafish (*Danio rerio*) [5]. Zebrafish show high homology in genes and organs with humans, their embryos have fast external development, optical clarity and are of a small size, all of which make them a great model for developmental and pathophysiological studies [6]. Zebrafish intestine closely resemble the mammalian small intestine anatomy and functions, though it is simpler and lacks a definite stomach. Zebrafish have been widely used for the study of tissue damage with leukocyte recruitment thanks to their highly conserved immune system and their transparency that allows live microscopy analysis [7]. In addition, zebrafish bone tissue is also very similar to that of humans for the composition of the mineralized matrix and for the presence of functional osteoblasts and osteoclasts [8]. Zebrafish larvae osteogenesis is characterized by the following two different types of ossification: endochondral ossification, that takes place from a cartilaginous scaffold, and intramembranous ossification, that is a direct ossification operated by mesenchymal stem cell precursors [9]. The neurocranium and the pharyngeal arches are ossified by endochondral processes, while vertebral bodies are formed by intramembranous ossification in a progressive cranio-caudal direction through notochord direct mineralization [10]. There are many models of zebrafish intestinal inflammation [11,12,13]. This study focused on the effects on zebrafish larval development when fed with a diet based on soybean flour, especially focusing on its effects on bone mineralization. Soybean flour is well known to induce several biological effects [14] including intestinal inflammation that has been already studied in various fish models [15,16,17,18] even in zebrafish larvae [19] and adults [20]. The model we used in this investigation was based on soybean meal (SBM) and was previously studied by Hedrera et al. who reported that zebrafish larvae already start to develop intestinal inflammation 2 days after the start of the SBM diet, because of its high saponin content [19]. Saponins are steroid or triterpenoid glycosides with several biological effects including alteration of the nutrient uptake through the intestinal membrane and the induction of inflammatory processes in the distal intestine characterized by infiltration in the lamina propria of immunoglobulin M (IgM) and T cells, eosinophils, macrophages, neutrophils and lymphocytes [14]. Therefore, the aim of this study was to analyze zebrafish larvae osteogenesis, with a focus on both endochondral and intramembranous ossification under the influence of a soybean diet that generates intestinal inflammation.

## 2. Results

### 2.1. SBM-Diet Induced Alterations in Mid-Intestine of Zebrafish Larvae

**Figure 1:**
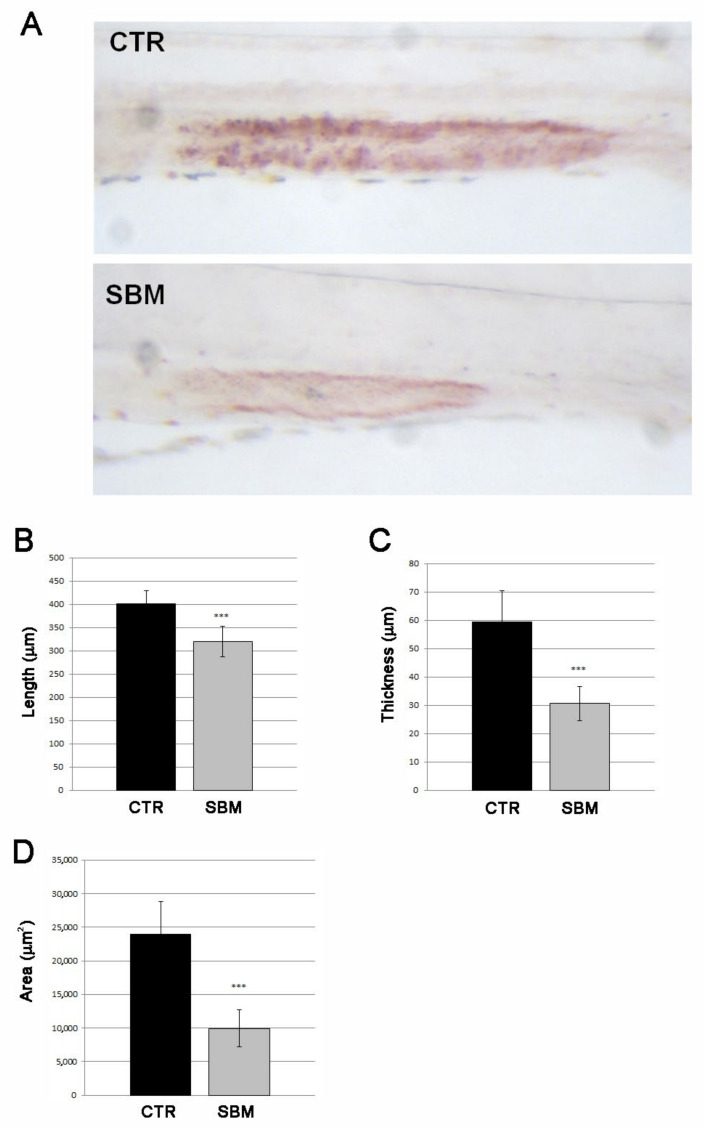
Intestinal effects of SBM. (**A**) Neutral red live staining of mid-intestine in larvae alimented with soy (SBM) or normal diet (CTR) showed decreased intensity of the staining in SBM larvae. Measure of length (**B**, SBM vs. CTR, *p* < 0.001), thickness (**C**, SBM vs. CTR, *p* < 0.001) and area (**D**, SBM vs. CTR, *p* < 0.001) of neutral red staining in controls (CTR) and soy (SBM). Data derived from 10 CTR and 10 SBM larvae, with tests performed 3 times as independent experiments with comparable results and significant differences were evaluated using the Student *t*-test (*** *p* < 0.001).

To induce dietary intestinal inflammation, zebrafish embryos were raised to 5 days post fertilization (dpf) and then fed with control diet (CTR) or SBM diet up to 9 dpf, as described by Hedrera [19]. Mortality rate, length and morphology remained unchanged between SBM and CTR larvae (data not shown). To confirm the presence of intestinal dysfunction, a live staining of lysosomal activity in the mid-intestine with neutral red was performed. A strong reduction in the neutral red staining was detected in SBM larvae compared to CTR, evaluated as intensity (Figure 1A), length (Figure 1B), thickness (Figure 1C) and area (Figure 1D).

### 2.2. Chondrogenesis as Well as Endochondral Ossification Were Not Affected in the Head under SBM-Diet

**Figure 2:**
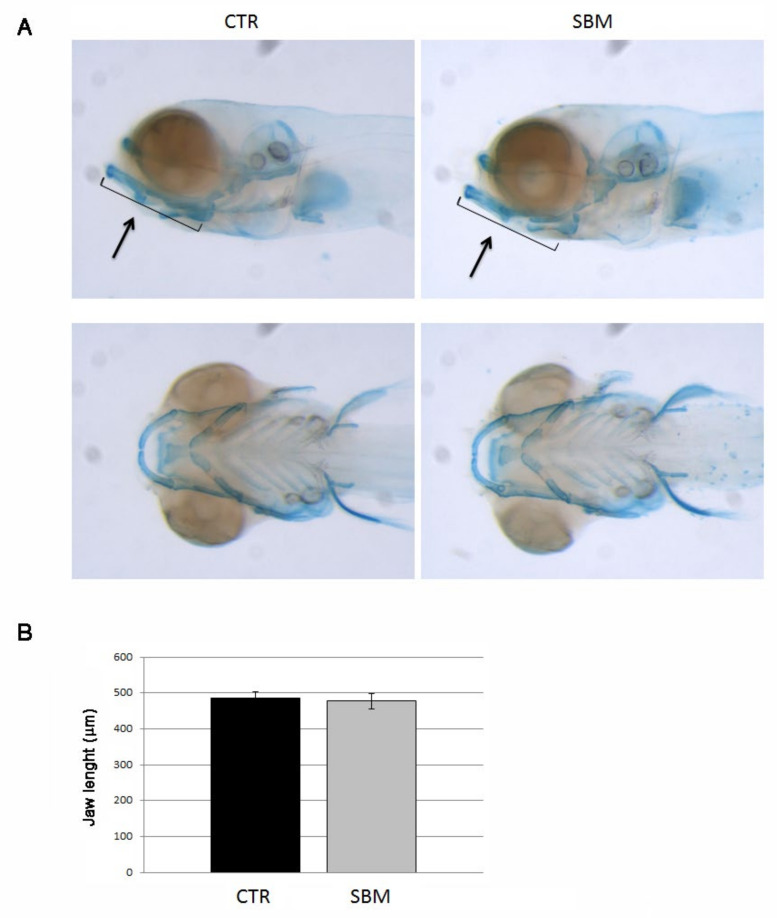
Head of zebrafish embryos stained with alcian blue. (**A**) Cartilage structures of CTR and SBM embryos visualized as lateral view (upper panel) and bottom view (lower panel). Black arrow indicates the jaw. (**B**) Length of jaw in CTR and SBM embryos. The measure was performed as indicated by black arrow in upper panel of A. Data derived from 10 CTR and 10 SBM larvae, with tests performed 3 times as independent experiments with comparable results. No significant difference was detected using the Student *t*-test.

To investigate the role of the intestine in skeletal development, we analyzed chondrogenesis and different patterns of bone mineralization in the head of developing larvae treated or not with SBM diet. The analysis of the chondrogenesis did not reveal significant differences between CTR and SBM larvae. In fact, SBM larvae stained with alcian blue showed a normal development of the cartilaginous structures at 9 dpf (Figure 2A) as confirmed by jaw measurements (Figure 2B), compared to CTR.

**Figure 3:**
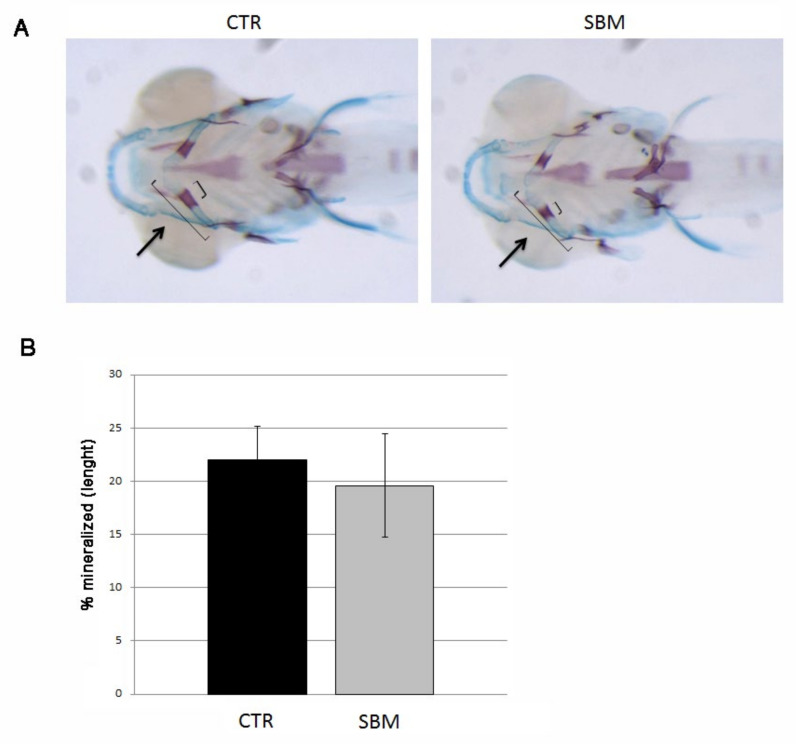
Bottom view of zebrafish larvae head double stained with alizarin red and alcian blue. (**A**) Mineralizing cartilage structures of CTR and SBM embryos can be visualized in purple, black arrow indicates the mineralizing area of cerathoyal. (**B**) Percentage of cerathoyal mineralization (small square bracket in **A**) compared to total length of jaw (long square bracket in **A**) in CTR and SBM larvae. Data derived from 10 CTR and 10 SBM larvae, with tests performed 3 times as independent experiments with comparable results. No significant difference was detected using the Student *t*-test.

To test endochondral mineralization, we performed double staining with alizarin red and alcian blue and we measured cerathoyal bone mineralization in the head. A slight slowdown of cerathoyal endochondral mineralization was detected in SBM larvae (Figure 3A) even if not statistically significant (SBM vs. CTR, −11%, Figure 3B).

### 2.3. Intramembranous Ossification Was Delayed in Head and Trunk of SBM Larvae

**Figure 4:**
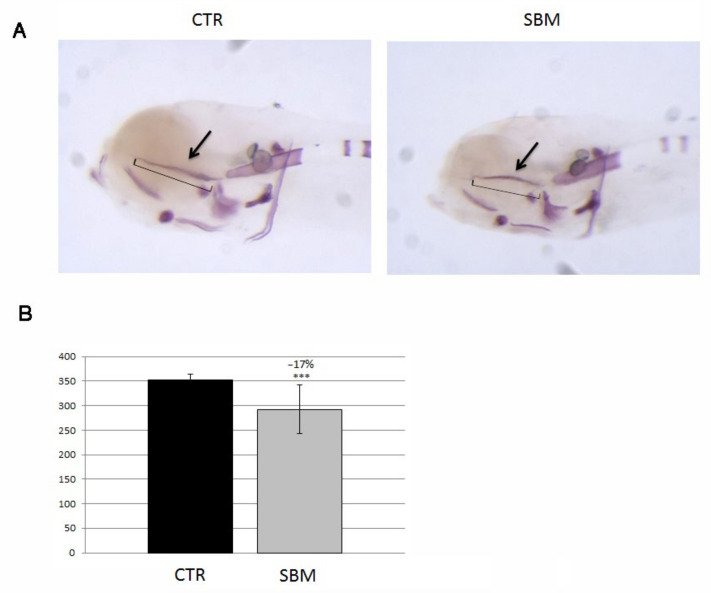
Later view of zebrafish larvae head stained with alizarin red. (**A**) Mineralizing structures of CTR and SBM embryos can be visualized in purple, black arrow indicates the ethmoid plate, which is formed by intramembranous mineralization. (**B**) Length of ethmoid plate (square bracket in **A**), measured in CTR and SBM larvae (SBM vs. CTR, −17%, *p* < 0.001). Data derived from 10 CTR and 10 SBM larvae, with tests performed 3 times as independent experiments with comparable results and significant differences were evaluated using Student’s *t*-test (*** *p* < 0.001).

SBM larvae stained only with alizarin red S (Figure 4A) displayed, in the head, a decreased intramembranous ossification of the ethmoid plate (SBM vs. CTR, −17%, *p* < 0.001, Figure 4B).

**Figure 5:**
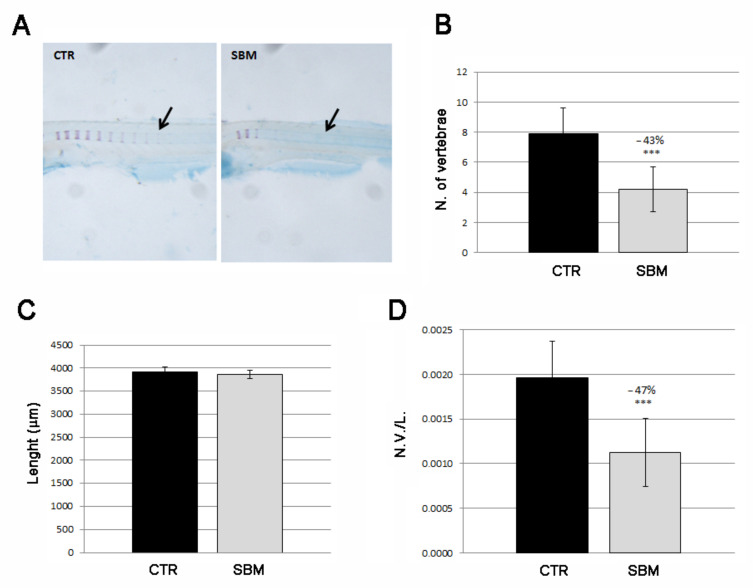
Lateral view of zebrafish larvae trunk double stained with alizarin red and alcian blue. (**A**) Mineralizing vertebrae of CTR and SBM embryos can be visualized in purple. Black arrow indicates a single vertebral body, which is formed by intramembranous mineralization. (**B**) Number of vertebrae in CTR and SBM larvae (SBM vs. CTR, −43%, *p* < 0.001). (**C**) Larval length. (**D**) Number of vertebrae/total body length (N.V./L.) (SBM vs. CTR, −47%, *p* < 0.001). Data derived from 10 CTR and 10 SBM larvae, with tests performed 3 times as independent experiments with comparable results and significant differences were evaluated by using Student’s *t*-test (*** *p* < 0.001).

We also analyzed the intramembranous ossification in the trunk focusing on vertebral mineralization (Figure 5A). CTR larvae showed an average of eight mineralized vertebrae at 9 dpf while SBM vertebral mineralization results almost halved (SBM vs. CTR, −43%, *p* < 0.001, Figure 5B). Total lengths of the larval body were taken to verify the normal general development of the body (Figure 5C) and were used to calculate a normalized index of vertebral mineralization (number of vertebrae/total body length, N.V./L.), thereby confirming the decreased SBM vertebral mineralization rate (SBM vs. CTR, −47%, *p* < 0.001, Figure 5D).

## 3. Discussion

The zebrafish gastrointestinal system is a good model to study physiological digestive processes and IBD pathogenesis because it is closely related to mammalian small intestines, both in terms of cellular anatomy and functions such as nutrients absorption and immunes functions [11,21].

The larval length analysis highlighted that SBM does not influence the general embryo development but induces intestinal region-specific effects. Neutral red is a staining method that can be easily used live in zebrafish larvae because it can diffuse across cellular membranes at pH 7, accumulating in acidified lysosomes and that can be protonated if exposed at an acidic pH [13]. This stain marks the lysosome-rich enterocytes (LREs) that are highly endocytic and that can be found in the zebrafish mid-intestine [22]. In fact, the zebrafish intestine is divided into three different segments named the intestinal bulb, which is the anterior part, the mid-intestine and posterior-intestine. In particular, the mid-intestine presents goblet cells and enterocytes [21] containing lysosomes that make this part of the intestine positive to neutral red staining [13]. In the zebrafish embryo enterocolitis model with trinitrobenzene sulfonic acid (TNBS), a significant reduction in intensity and the extension of neutral red staining associated with inflammation and loss of function were observed [13,23]. Similarly, in our model with zebrafish larvae fed with SBM from 5 to 9 dpf, the neutral red mid-intestine staining results were less intense compared to the control intestines. These data suggest that the zebrafish larval intestine pH decreases and its function is altered following a soy-based diet.

Hedrera et al. [19] also found an increase in pro-inflammatory cytokines, Interleukin 1 beta (IL-1β) and Interleukin 8 (IL-8) in the same model. Another study performed on 6–9 dpf zebrafish larvae highlighted that saponin exposure in water increases the number of neutrophils present in the intestinal area in a dose dependent manner and indices of immune stimulation, which were detected as am increased pro-inflammatory cytokine expression [12].

IBD is often related to bone health issues such as low bone mineral density, osteoporosis and osteopenia [4] through different mechanisms such as intestinal vitamin D calcium malabsorption and the involvement of cytokines in the intestinal inflammatory process [24] that directly promote bone loss [25]. Our data highlight that SBM affects zebrafish larvae mineralization with a very specific effect related only to intramembranous mineralization. In fact, endochondral ossification does not seems to be affected by SBM since the cartilaginous head tissue develops normally and also its subsequent mineralization is normal. On the contrary, head and vertebral intramembranous ossification is reduced by SBM. Specific gene expressions mediated by Runt-related transcription factor 2 (Runx2) drive osteoblast differentiation from osteochondral progenitor stem cells [26]. Several *in vitro* experiments demonstrated that Tumor necrosis factor—alpha (TNF-α) inhibits osteoblast differentiation at the early stage of differentiation [27,28]. The intestine and its microbiome have an essential role in the regulation of bone health principally through the regulation of calcium absorption. Numerous studies have been performed concerning the modulation of the gut microbiome on various animal models such as rodents, chicken and even zebrafish [29] Microbiota play essential roles in the development of zebrafish embryos, influencing the immune system, physiology, reproduction, nutrients metabolism and ossification [30]. The importance of zebrafish microbiota on embryo mineralization [30] suggests that any alteration can influence, positively or negatively, early mineralization processes. It has been previously demonstrated that a probiotic-supplemented diet effects the growth and calcification of zebrafish larvae due to the probiotic capability to modulate the transcription of 212 genes including genes implicated in nutrients metabolism [31]. Avella et al. demonstrated that the administration of *Lactobacillus rhamnosus* anticipated backbone development in correlation with insulin-like growth factor (IGF) system stimulation while involving vitamin D and retinoic acid, key factors for chondrogenesis and morphogenesis [30] as well as the stimulation of the expression of other key genes for ossification [32]. As is the case in humans and mice, zebrafish enterocolitis is closely related to the microbiota. TNBS-exposed zebrafish larvae demonstrated that antibiotic treatment diminished the inflammation and prevented the transcription of pro-inflammatory cytokines [13]. Oehlers et al. performed another study based on dextran sodium sulfate (DSS) induced enterocolitis, finding that this model was also microbiota dependent with neutrophilic inflammation, which can be ameliorated with antibiotic and anti-inflammatory treatments [33]. López Nadal [12] performed the microbial sequencing of whole zebrafish larvae and highlighted that saponin exposure modified microbial composition, thereby increasing its diversity, which has also been observed in studies with other fish [34,35]. These microbiota alterations due to saponin exposure in SBM can contribute to the alterations in larval mineralization. Our data highlighted that a specific alteration in zebrafish intramembranous larval mineralization correlated with SBM diet, which interestingly does not affect endochondral mineralization. Diet’s influence on the intestinal microbiome should be considered in future studies to better understand the effects of soybean meal on zebrafish development. Possible limitations of the work are the differences in the digestive trait between humans and fish. Zebrafish, lacking a definite stomach, may have different digestive processes.

Our results confirm zebrafish larvae as excellent intestinal, inflammatory and mineralization models that can contribute to elucidating *in vivo* IBD-related bone disorders, thereby validating their use for large-scale drug tests.

## 4. Materials and Methods

### 4.1. Ethic Statement

This investigation was performed in the Zebrafish Laboratory (IRCCS R. Galeazzi, GSD Foundation, Milan, Italy) according to Italian and European guidelines on research (EU Directive 2010/63/EU). Zebrafish experimentation and all protocols of this study were approved by Ministry of Health (Italy) with authorization n. 742/2019-PR.

### 4.2. Animals

*Danio rerio* of AB strain were housed in ZEBTEC^©^ Bench Top System (Tecniplast, Buguggiate, Italy) and maintained at 28 °C under standard conditions [36]. Embryos were obtained with a single pair of adults and were checked for general health conditions under a light stereomicroscope as described by Kimmel et al. [37].

### 4.3. Treatments

Embryos were maintained at 28 °C in a dark incubator in a standard growing medium (E3 medium, 5 mM NaCl, 0.17 mM KCl, 0.33 mM CaCl_2_, 0.33 mM MgSO_4_) for up to 5 days post fertilization (dpf) and were then moved to fish tanks with E3 medium and fed up to 9 dpf with standard fish diet (CTR, Baby microgranules, SHG, Ovada, Italy) or soybean diet (SBM). SBM was formulated based on a previously published study performed on zebrafish larvae [19] using 50% *w*/*w* standard diet and 50% *w*/*w* soybean flour type I (Sigma Aldrich, St. Louis, MO, USA), a defatted soybean flour with a high protein content (protein ~ 52% (85+% dispersible and 1% fat) that ensures a very high saponin content. A total of 40 larvae were fed with each diet and the entire experiment was repeated 3 times for a total amount of 240 larvae.

### 4.4. Histochemical Analysis

At the end of the treatment (9 dpf), 10 larvae of each diet were incubated in 2.5 μg/mL neutral red E3 medium live staining solution at 28 °C for 30 min in the dark as described by Herbomel et al. [38]. Then, larvae were repeatedly washed in E3 medium and anaesthetized with 0.01% tricaine methanesulfonate (Sigma Aldrich, St. Louis, MO, USA) E3 medium solution.

The 30 remaining larvae of each diet were euthanized using a 300 mg/L tricaine methanesulfonate (Sigma Aldrich, St. Louis, MO, USA) solution [39] and fixed in 3.5% formaldehyde/0.1 M sodium phosphate buffer. Then, Alcian Blue 8GX (Sigma Aldrich, St. Louis, MO, USA) and/or Alizarin red S (ARS, Sigma Aldrich, St. Louis, MO, USA) double acid-free staining [40] were performed to stain, respectively, cartilage and bone tissue. Both the double staining and the two single stainings were performed, each staining on 10 larvae, to better highlight bone and cartilage tissue separately.

All larvae were examined under a light/fluorescence stereomicroscope (SZX-ZB7 Olympus, Tokyo, Japan) with images acquired using a Discovery CH30 camera (Tiesselab, Milan, Italy). Images were analyzed with ISC Capture software (version 2.5) to perform the measurements. We analyzed alcian blue images to measure the jaw and alizarin red S images to measure the ethmoid plate. Using double staining, we measured the mineralization level of the cerathoyal (measured as length of the mineralized portion (positive for alizarin red S staining)*100/total length of the jaw (alcian blue staining) and the vertebral mineralization rate (N.V./L., calculated as number of mineralized vertebral bodies (N.V., positive for alizarin red S staining) normalized for the length ((L.) for each larva).

### 4.5. Statistical Analysis

Data were derived from 40 larvae for each diet, 10 larvae were used for each analysis, and each test was performed 3 times as independent experiments with comparable results, using a total amount of 240 larvae. Data were used to calculate the mean value expressed as mean of the means of the 3 independent experiments ± standard deviation versus control. Data were plotted on SigmaStat software (version 3.5) (San Jose, CA, USA) and subjected to Student’s *t*-test with the significance values set at *p* < 0.05 (*), *p* < 0.01 (**) and *p* < 0.001 (***).

## Data Availability

Data are contained within the article.
